# Oral Immunization of Chickens with Probiotic *Lactobacillus crispatus* Constitutively Expressing the α-β2-ε-β1 Toxoids to Induce Protective Immunity

**DOI:** 10.3390/vaccines10050698

**Published:** 2022-04-29

**Authors:** Mohammad Zeb Khan, Fengsai Li, Xuewei Huang, Muhammad Nouman, Roshna Bibi, Xiaolong Fan, Han Zhou, Zhifu Shan, Li Wang, Yanping Jiang, Wen Cui, Xinyuan Qiao, Yijing Li, Xiaona Wang, Lijie Tang

**Affiliations:** 1 College of Veterinary Medicine, Northeast Agricultural University, Harbin 150030, China; khan2022@126.com (M.Z.K.); yilvwenrou@126.com (F.L.); huangxuewei126@126.com (X.H.); fxl13796687571@163.com (X.F.); zhouhan9659@163.com (H.Z.); shanzhifu@126.com (Z.S.); wanglicau@163.com (L.W.); jiangyanping2017@126.com (Y.J.); cuiwen_200@163.com (W.C.); qiaoxinyuan@126.com (X.Q.); yijingli@163.com (Y.L.); 2 Khyber Medical College, Peshawar 25120, Pakistan; nouman2022@126.com; 3 Department of Boyany, University of Swat, Mingora 19200, Pakistan; bibi2022@126.com; 4 Heilongjiang Key Laboratory for Animal Disease Control and Pharmaceutical Development, Harbin 150030, China

**Keywords:** *Lactobacillus crispatus*, *Clostridium perfringens*, α-β2-ε-β1 toxoid, oral immunization, protective effect

## Abstract

*Clostridium perfringens* (*C. perfringens*) is a bacterium that commonly causes zoonotic disease. The pathogenicity of *C. perfringens* is a result of the combined action of α, β, and ε exotoxins. In this study, *Lactobacillus crispatus* (pPG-T7g10/*L. crispatus*) expressing the main toxoids of *C. perfringens*, α, ε, β1, and β2, with EGFP-labeling, was constructed, and the protective effect was estimated in chickens. The α-β2-ε-β1 toxoid was constitutively expressed for confirmation by laser confocal microscopy and western blotting, and its immunogenicity was analyzed by enzyme-linked immunosorbent assay (ELISA) and immunohistochemical assays. After booster immunization, the probiotic vaccine group showed significantly higher levels (*p* < 0.05) of specific secretory IgA (sIgA) and IgY antibodies in the serum and intestinal mucus. Furthermore, the levels of cytokines, including interferon (IFN)-γ, interleukin (lL)-2, IL-4, IL-10, IL-12, and IL-17, and the proliferation of spleen lymphocytes in chickens orally immunized with pPG-E-α-β2-ε-β1/*L. crispatus* increased significantly. Histopathological observations showed that the intestinal pathological changes in chickens immunized with pPG-E-α-β2ε-β1/*L. crispatus* were significantly alleviated. These data reveal that the probiotic vaccine could stimulate mucosal, cellular, and humoral immunity and provide an active defense against the toxins of *C. perfringens*, suggesting a promising candidate for oral vaccines against *C. perfringens.*

## 1. Introduction

*Clostridium perfringens* (*C. perfringens*) is a common zoonotic pathogen that is abundantly present in the environment. It often causes gas gangrene, necrotizing enteritis, and enterotoxemia in cattle, sheep, and chickens [1,2]. The exotoxin secreted by *C. perfringens* is the main pathogenic factor that infects animals, causing rapid onset, high mortality, and serious economic losses to animal husbandry [3]. The pathogenicity of *C. perfringens* results from the synergy of multiple exotoxins, of which α, β, and ε are the most common and highly toxic [4]. These toxins can result in a variety of serious diseases in livestock and poultry, of which enterotoxemia and necrotic enteritis are common [2,3]. In chickens, *C. perfringens* promotes the development of necrotizing enteritis, leading to a mortality rate of up to 50% [5]. Furthermore, it causes chronic damage to the intestinal mucosa (subclinical infection), which is accompanied by poor mental status and weight loss, resulting in severe economic losses [6]. Therefore, the development of an operative vaccine against *C. perfringens* infections is highly desirable. Currently, many countries use antibiotics and sulfonamides to prevent and treat diseases caused by *C. perfringens* infection in animals [7]. Because of the rapid onset and course of the disease, large-scale broad-spectrum antibiotics are used. However, growing attention is being paid to the harm caused by the extensive use of antibiotics in humans and animals [8]. Therefore, to limit the spread of the disease and reduce large economic losses for the livestock industry, it is necessary to adopt practical measures to prevent and control such diseases [9].

As *C. perfringens* mainly infects the intestinal tract of animals, developing an oral immunization vaccine is a promising approach to prevent the occurrence of this disease. Lactic acid bacteria (LAB) are Gram-positive bacteria which are widely distributed in animal intestinal tissues and play a key role in maintaining the stability of microbial ecology and inducing mucosal and systemic immunity [10]. Use of *Lactobacillus*, a member of LAB, has many advantages: it has been proven to be a food-grade safe microorganism for application in the research of food-grade gene cloning vector systems and expression vector systems; it interacts with cells and induces the production of a series of cytokines; and it has the ability to tolerate bile acids and is affected by immune adjuvants [11]. It helps in protecting various mucosal structures by colonizing the system to maintain the microecological stability of the flora through mucosal immunity and generation of the sIgA antibody [12]. *Lactobacillus* induces a mucosal immune response in the body. Some of these microorganisms pass through the microfold cells between the epithelial cells of the small intestine, whereas some pass through the dendritic cells under the epithelial cells to enter the lamina propria of the small intestine tissue, where they activate Th2 lymphocytes through the activation of plasma cells, which secrete specific IgA antibodies [13,14]. Specific IgA antibodies can interact with secretory fragments produced by the intestinal mucosal epithelium on the surface of the intestinal mucosa to prevent adhesion of pathogenic bacteria and invasion of mucosal tissues [15,16]. Specific lymphocytes then dissociate in the mucosal lamina propria, glands, and other reactive sites to trigger an immune response at multiple mucosal sites [17]. With continuous improvements in research technology, it is now possible to obtain genetically engineered *Lactobacillus* with better antigenic quality, whose application in production practices has far-reaching significance. Song et al. developed a stable and marker-free *Lactobacillus* strain, realizing expression of the alpha-toxin gene of *Clostridium perfringens* on the surface of bacteria [18]. Gao et al. constructed a genetically engineered *Lactobacillus casei* (pPG-α/*L. casei* 393) for constitutively expressing the toxoid of *C. perfringens* α-toxin, a BLA/c mice experiment to evaluate the oral immunogenicity of pPG-α/*L. casei* 393, the results indicating that the strain could induce the production of mucosal, humoral, and cellular immunity, and provide a protective effect to a certain degree [19].

In this study, the recombinant strain pPG-T7g10/*L. crispatus*, expressing the main toxoids of *C. perfringens*, α, β1, ε, and β2, with non-antibiotic resistance, was constructed, and the genes were modified by our laboratory for mutating the 68th histidine residue to a glycine residue in α toxin, the 234th cysteine residue to a glycine residue in β2 toxin, and the 106th histidine residue to a proline residue in ε toxin. Its immunogenicity in chickens was evaluated for its protective effect against *C. perfringens* infection.

## 2. Material and Methods

### 2.1. Strains and Plasmid

The *L. crispatus* N-11 strain was isolated from the intestine of a chicken in our laboratory [20] and grown in de Man, Rogosa, and Sharpe (MRS) broth (Sigma, St. Louis, MO, USA) at 37 °C without shaking. *C. perfringens* types A (C57-1), B (CCVC-81), and C (CACC-61) were obtained from the China Institute of Veterinary Drug Control, Beijing, China. *Escherichia coli* strain JM109 and the engineered strain pPG-T7g10/TG1, containing the constitutively expressed plasmid were cultivated in our laboratory at 37 °C in Luria–Bertani (LB) broth with shaking. The pMD19-EGFP plasmid containing the gene encoding EGFP (Accession No. U57607) and the pMD18-T-α-β2-ε-β1 plasmid, which had been constructed to contain a fusion of the genes encoding the quadruple toxoid of *C. perfringens* α (H68G), β_2_ (C234G), ε (H106P), and β_1_ (C292A, D81A, and K83A), were preserved in our lab [11,21]. The pPG-T7g10-PPT plasmid, which contains the HCE strong constitutive promoter, T7g10 enhancer, and PgsA anchor sequence along with the rrnBT1T2 terminator, was constructed in our laboratory [21,22]. The present study was conducted in accordance with the guidelines for the Care, Maintenance, and Use of Laboratory Animals of the National Institute of Health. The protocol was approved by the Ethical Committee for Animal Experimentation of the Northeast Agriculture University, China.

### 2.2. Construction of a Recombinant Expression System in L. crispatus

The recombinant strain was constructed using a previously described method [23]. Briefly, the gene encoding the α, β2, ε, and β1 toxoids of *C. perfringens* was acquired from the plasmid pMD19-T-α-β2-ε-β via PCR amplification and then inserted into the constitutive expression vector pPG-T7g10-PPT, generating the recombinant plasmid pPG-α-β2-ε-β1. Subsequently, the fragment encoding EGFP was inserted upstream of the fusion genes α, β2, ε, and β1 and fused with α-β2-ε-β1 by a linker, resulting in creation of the plasmid pPG-E-α-β2-ε-β1. Finally, the recombinant plasmid pPG-E-α-β2-ε-β1 was electroporated into *L. crispatus* N-11 competent cells and the positive strain was validated using laser confocal microscopy, flow cytometry, and western blotting.

### 2.3. Toxin Genes Expression Analysis

pPG-E-α-β2-ε-β1/*L. crispatus* N-11 was cultured in MRS medium at 37 °C for 16 h, centrifuged at 10,000× *g* for 5 min, and washed three times with sterilized phosphate-buffered saline (PBS; pH = 8.0). The precipitate was incubated with lysozyme and ultrasonicated. Then, 2 × sodium dodecyl sulfate (SDS) buffer solution was added and the sample was boiled for 10 min. After boiling, the sample was centrifuged, and the supernatant was collected. The supernatant was then subjected to SDS-polyacrylamide gel electrophoresis (PAGE) in a 10% gel, followed by western blot analysis using an anti-α toxin polyclonal and mouse anti-toxoid monoclonal antibody (generated in our laboratory) at a dilution ratio of 1:500. Subsequently, the membrane was incubated with the secondary antibody horseradish peroxidase (HRP)-conjugated goat anti-mouse IgG (Sigma, USA) diluted 1:5000. Immunolabeled bands were visualized using chemiluminescent substrate reagent (Pierce, Rockford, IL, USA).

To further analyze the protein expressed on the cell surface of pPG-T7g10-PPT/*L. crispatus* N-11, the recombinant pPG-E-α-β2-ε-β1/*L. crispatus* N-11 was cultured in MRS medium at 37 °C for 16 h, centrifuged at 10,000× *g* for 5 min, washed three times with sterilized PBS, resuspended in sterile PBS, and then the cells were examined using laser confocal microscopy [19].

### 2.4. Colonization Capability of the Recombinant Strain

For the colonization test, recombinant pPG-E-α-β1-ε-β2/*L. crispatus* N-11 grown until the optical density at 600 nm (OD600) was approximately 1.0, and then centrifuged at 5000× *g* for 10 min. The pelleted cells were resuspended at a concentration of 10^10^ colony-forming units (CFU)/mL and then washed twice with sterile PBS. The strain was then labeled with carboxyfluorescein diacetate-succinimidyl ester (CFDA-SE) and incubated for 20 min at 37 °C. Subsequently, the cells were washed and resuspended in PBS, and the labeling ratio was determined using flow cytometry. A total of 15 chickens were inoculated with CFDA-SE-labeled recombinant strain (approximately 10^9^ CFU/mL). Intestinal segments of the jejunum, ileum, and colon were collected on days 1, 4, 7, 11, and 15 (*n* = 3), and the colonization capability of the recombinant strain in the intestinal tract was assessed using flow cytometry.

### 2.5. Immunization

The maintenance and use of the experimental animals followed the appropriate international (OIE Terrestrial Animal Health Code) and national guiding principles (CNAS-CL 06:2018). In this study, to evaluate the immunogenicity of the engineered strain pPG-E-α-β2-ε-β1/*L. crispatus* N-11 as oral vaccine, 1-day-old specific pathogen-free (SPF) chickens provided by the Harbin Veterinary Institute were selected as an animal model and kept under SPF environments with free access to standard feed and water. The basal feed diet ingredients and environmental factors of all the groups were equally managed. Before oral immunization, the recombinant strain pPG-E-α-β2-ε-β1/*L*. *crispatus* N-11 was cultured to an OD_600_ = 1.0, washed with PBS, and resuspended to a concentration of 1 × 10^10^ CFU/mL in PBS. The immunization procedure was as follows. SPF chickens were divided into five groups. The recombinant strain group (*n* = 36) was orally immunized with 1 mL pPG-E-α-β2-ε-β_1_ *L. crispatus* N-11; the empty carrier vector group (*n* = 36) was immunized with 1 mL of pPG-T7g10-PPT/*L. crispatus* N-11; the control group (*n* = 36) was orally inoculated with 200 μL of *L. crispatus* N-11; the mock control group (*n* = 36) received 200 μL of PBS, and this group was considered as a control without inoculation (*n* = 15). All chickens were immunized orally, and the immunization schedule was repeated three times at 14-day intervals. Each dose was administered (offered) once every three days. The samples were collected on days 0, 7, 14, 21, 28, 35, and 42 [19].

### 2.6. Enzyme-Linked Immunosorbent Assay (ELISA)

Serum and intestinal mucus samples were collected from three chickens randomly in each group on days 0, 7, 14, 21, 28, 35, and 42 after primary immunization, and specific antibody IgA and IgY levels were detected using ELISA as described earlier [10,19]. Briefly, purified α-β2-ε-β1 toxoid protein (5 ng/mL) was added to 96-well polystyrene microtiter plates and incubated overnight at 4 °C, after which the plates were washed three times with PBST. The plates were then incubated for 2 h with 200 μL of 5% skim milk at 37 °C. Next, the collected serum and intestinal mucus samples (sera diluted at 1:50 and supernatant of intestinal mucus diluted at 1:10) were added to each reaction well (100 μL per well) as primary antibodies and incubated at 37 °C for 1 h, followed by washing three times with PBST. HRP-labeled goat anti-chicken IgY (Sigma, USA) or IgA antibody (Sigma, USA) diluted at 1:5000 were used as secondary antibodies. Next, 100 μL of o-phenylenediamine dihydrochloride (Sigma, USA) color developing solution was added to each well and incubated at 37 °C for 15 min, and the absorbance at OD490 was measured using a microplate reader (BioTek, Winooski, VT, USA) after adding the stop solution (2 M H_2_SO_4_). On day 35 post-immunization, the levels of interferon (IFN)-γ, interleukin (lL)-2, IL-4, IL-10, IL-12, and IL-17 in the samples were determined using an ELISA kit (Biosource International, Camarillo, CA, USA) according to the manufacturer’s guidelines.

### 2.7. Immunohistochemical Analysis

On day 42 post-immunization, intestinal samples from the duodenum, jejunum, ileum, and cecum (1 cm^2^) were obtained from chickens immunized with pPG-E-α-β2-ε-β1/*L. crispatus* N-11 and the PBS control group (inoculation-free group). Collected samples were fixed with 10% neutral-buffered formalin, processed with paraffin wax, and cut to a thickness of 5 μm. Immunoglobulin (IgA) cells confined to the intestine were detected using immunohistochemistry. Immunohistochemical staining and calculations were performed as described previously [24]. Polyclonal mouse anti-chicken IgA (Invitrogen, Carlsbad, CA, USA) and biotinylated goat anti-mouse IgG (ZSGB-BIO, Beijing, China) were used as the primary and secondary antibodies, respectively.

### 2.8. Lymphocyte Proliferation in Immunized Chickens

On day 42 post-immunization, splenocytes were collected from chickens in each group and used in the lymphocyte proliferation assay conducted using the respective kit from Sigma Life Science (Histopaque-1077 sterile-filtered solution, density: 1.077 g/mL). Briefly, 100 μL of spleen cell suspension (5 × 10^6^ cells/mL) was incubated in each well of a 96-well plate (the cell culture was composed of 90% RPMI 1640 medium and 10% fetal bovine serum) at 37 °C in a 5% CO_2_ atmosphere for 8–12 h. Cells were then stimulated with purified recombinant α-β2-ε-β1 protein at final concentrations of 0.01, 0.1, 1, and 10 mg/mL for 60 h, and an equal volume of the medium was added to the control cells. Then, 10 µL of thiazolyl blue tetrazolium bromide (MTT) at 5 mg/mL was added to each well and incubated at 37 °C for 5 h. Finally, the absorbance was measured at 600 nm and the stimulated index was estimated as follows: SI = OD600 (sample)/OD600 (blank control).

### 2.9. Protection Effect

To estimate the immune protection of chickens orally vaccinated with pPG-E-α-β2-ε-β1/*L. crispatus* N-11, on day 42 post-immunization, chickens inoculated with pPG-E-α-β2-ε-β1/*L. crispatus* N-11, pPG-T7g10-PPT/*L. crispatus* N-11, *L. crispatus* N-11, and PBS were challenged with a typically lethal dose (LD_100_) of the natural α-β2-ε-β1 toxin combined with 10^8^ CFU of *C. perfringens* type A and type B oral pathogenic bacteria via oral administration using an oral gavage needle; the respective control group was not challenged.

The daily subsistence ratio of the experimental chickens in each group was counted post-challenge, and histopathological changes in the intestinal sections, including duodenum, jejunum, ileum, and cecum, of all the groups were observed, with special consideration given to the structure, color, and occurrences of necrosis, hemorrhage, congestion, and cell infiltration.

### 2.10. Analysis of Gut Microbiota through 16S rDNA Sequence

To evaluate the effect of the recombinant *L. crispatus* pPG-E-α-β2-ε-β1/*L. crispatus* N-11 on the gut microbiota, we divided a total of 24 chickens into three groups: recombinant *L. crispatus*-, *C. perfringens*-, and PBS-inoculated groups (*n* = 8). The intestinal contents were collected on days 7 and 14 from each group. Microbial DNA was extracted using a Tiangen Stool DNA kit (Biotech, Beijing, China), according to the manufacturer’s instructions. After extraction, the concentration was measured using a NanoDrop 2000 spectrophotometer. After extracting genomic DNA from the samples, the V3-V4 region of 16S rDNA was amplified using specific PCR primers (341F: CCTACGGGNGGCWGCAG; 806R: GGACTACHVGGGTATCTAAT). Purified primers were mixed in equal quantities and connected to the Illumina adapter and linker sequences. A paired-end sequencing (2 × 250) was performed with equal amounts of purified amplicons on the Illumina platform, then analysed using an Illumina HiSeq2500 from GeneDenovo (Guangzhou, China).

### 2.11. Statistical Analysis

All data conformed to a normal distribution for analyzing with Shapiro–Wilk test, shown as mean ± standard deviation (SD). All experiments were performed in triplicate. Statistical significance was evaluated via one-way analysis of variance (ANOVA) using Tukey’s multiple comparison test and Prism 7 software. ** *p* < 0.01 and * *p* < 0.05 indicated statistical significance.

## 3. Results

### 3.1. Identification of Protein Expression

**Figure 1:**
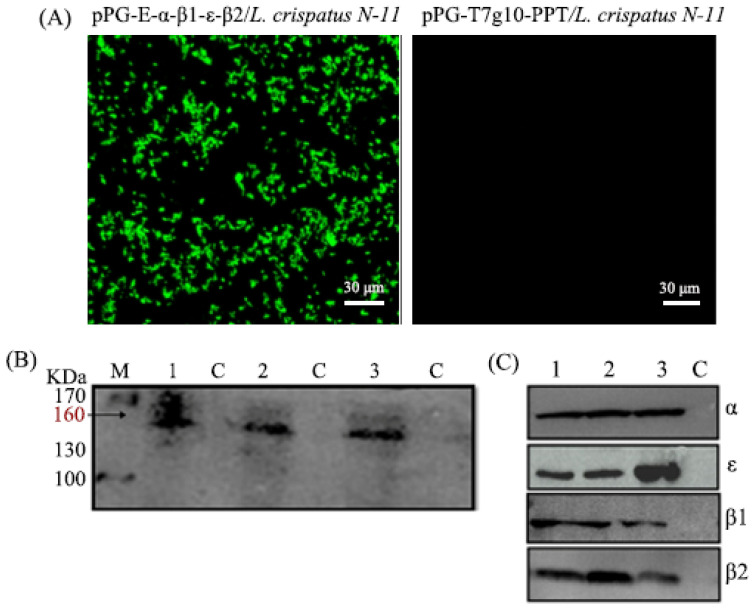
The constitutive expression of the toxoid proteins was evaluated using an ultra-high resolution microscope and western blotting. (**A**) The determination of the cell surface expression of α-β1-ε-β2 using laser confocal microscopy. Results show clear green fluorescence on the surface of the cells of pPG-E-α-β1-ε-β2/*L. crispatus* N-11, but not on the pPG-T7g10-PPT/*L. crispatus* N-11 strain. Western blotting with anti-α toxin polyclonal antibody, showing a band of expected size around 160 kDa (**B**) or mouse anti-α/β2/ε/β1 toxins monoclonal antibody (**C**). M: Pre-stained protein Marker; 1-2-3: pPG-E-α-β1-ε-β2/*L. crispatus* N-11; C: empty vector.

The expression vector pPG-T7g10, which is a cell surface expression system, was used to analyze cell surface expression of the α-β2-ε-β1 toxoid protein with laser confocal observation, and the results showed that there were clear green fluorescent signals on the cell surface of pPG-E-α-β2-ε-β1/*L. crispatus* N-11 cells, but not on the pPG/*L. crispatus* N-11 cells (Figure 1A). To further detect the fusion protein expression of the toxoid by the recombinant *L. crispatus* N-11 constructed in this study, the samples were assessed using western blotting analysis. A specific band was observed for the recombinant pPG-E-α-β2-ε-β1/*L. crispatus* N-11 (Figure 1B,C; Figures S1 and S2), but not for pPG-T7g10-PPT/*L. crispatus* N-11 or *L. crispatus*, suggesting that the fusion protein was expressed by the genetically engineered pPG-E-α-β2-ε-β1/*L. crispatus* N-11.

### 3.2. Intestinal Colonization Ability of Recombinant L. crispatus N-11

On days 1, 4, 7, 11, and 15, the jejunal, ileal, and colonic contents were scraped to assess the colonization capacity of pPG-E-α-β2-ε-β1/*L. crispatus* N-11 using flow cytometry. The results indicate that pPG-E-α-β2-ε-β1/*L. crispatus* N-11 could persist and adhere to the jejunum, ileum, and colon of chickens, as shown in Table 1, with colonization rates of 61%, 54%, and 57% on day 1, respectively. The colonization number of recombinant bacteria in the chickens gradually decreased over time along the internal environment of the digestive tract, with the colonization rates of the jejunum, ileum, and colon being 21%, 17%, and 19% on day 15, respectively.

### 3.3. Immunogenicity

**Figure 2:**
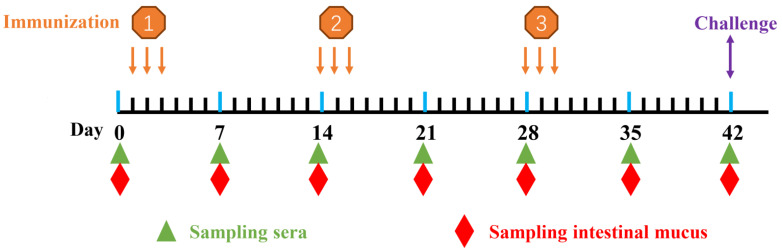
The oral immunization structure and schedule of sampling. The immunization was performed for consecutively three days with a 2-week interval between booster immunizations. The serum and intestinal mucus (*n* = 3) were collected on days 0, 7, 14, 21, 28, 35, and 42. The challenge test was carried out on day 42 post-immunization.

**Figure 3:**
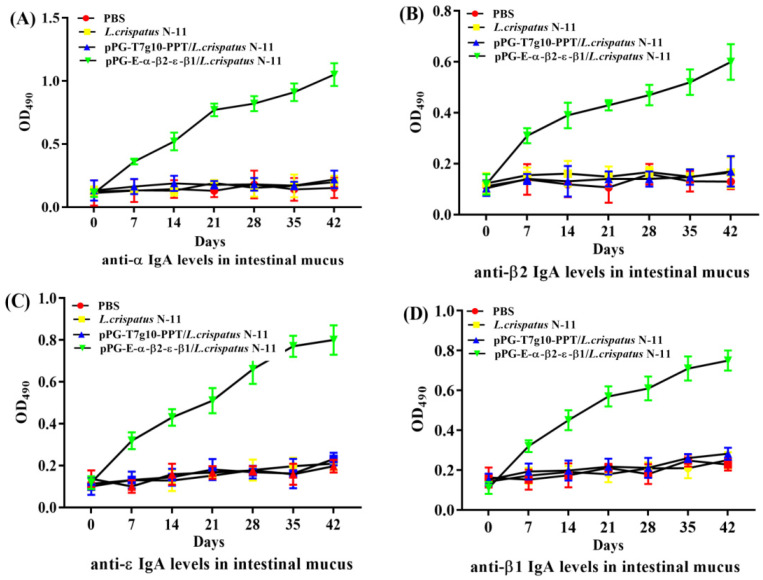
Antigen-specific IgA antibody levels in intestinal mucus of chickens immunized with pPG-E-α-β1-ε-β2/*L. crispatus* N-11 was analyzed using ELISA. The intestinal mucus samples (*n* = 3) were collected on days 0, 7, 14, 21, 28, 35, and 42 from each group of chickens. (**A**) Anti-α specific IgA; (**B**) anti-β2 specific IgA; (**C**) anti-ε specific IgA; (**D**) anti-β1 specific IgA. Data are presented as the mean ± standard error in each group.

**Figure 4:**
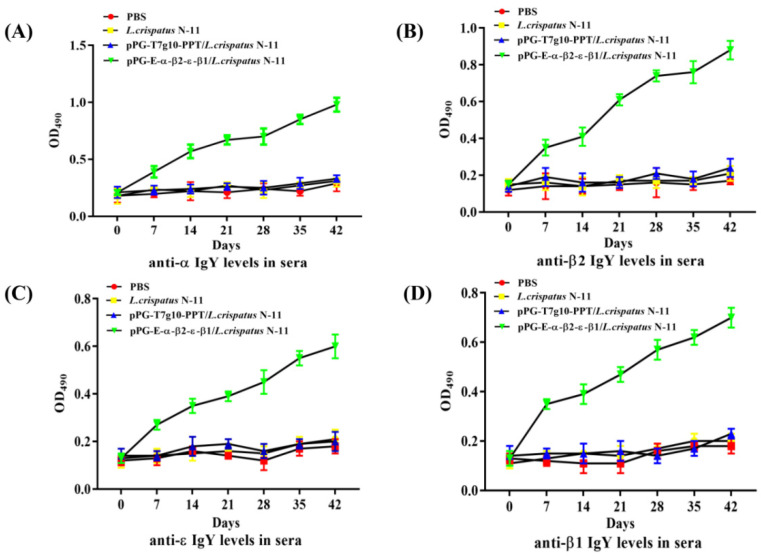
Antigen-specific IgY antibody level in sera of chickens immunized with pPG-E-α-β1-ε-β2/*L. crispatus* N-11 was analyzed using ELISA. The intestinal mucus samples (*n* = 3) were collected on days 0, 7, 14, 21, 28, 35, and 42 from each group of chickens. (**A**) Anti-α specific IgY; (**B**) anti-β2 specific IgY; (**C**) anti-ε specific IgY; (**D**) anti-β1 specific IgY. Data are presented as the mean ± standard error in each group.

To assay the immunity of chickens orally immunized with strain pPG-E-α-β2-ε-β1/*L. crispatus* N-11, the levels of specific anti-α-β2-ε-β2 toxoid IgA and IgY antibodies were detected using ELISA. The immunization scheme and sample collection are shown in Figure 2. According to the ELISA results, there were no significant differences (*p* > 0.05) in IgA and IgY antibody levels among the groups before immunization. However, after booster immunization, particularly on day 14 post-immunization, anti–α-β2-ε-β1 toxoid specific IgA level (Figure 3) in intestinal mucus and IgY antibody level (Figure 4) in sera of chickens in the pPG-E-α-β1-ε-β2/*L. crispatus* N-11 group were significantly higher (*p* ˂ 0.05) than those of groups “pPG/*L. crispatus*” *L. crispatus* N-11” and “PBS”. Conversely, there was no significant difference (*p* > 0.05) between the control groups before and after immunization. These results indicate that oral pPG-E-α-β2-β1/*L. crispatus* N-11 induced specific mucosal and systemic immune responses in chickens.

**Figure 5:**
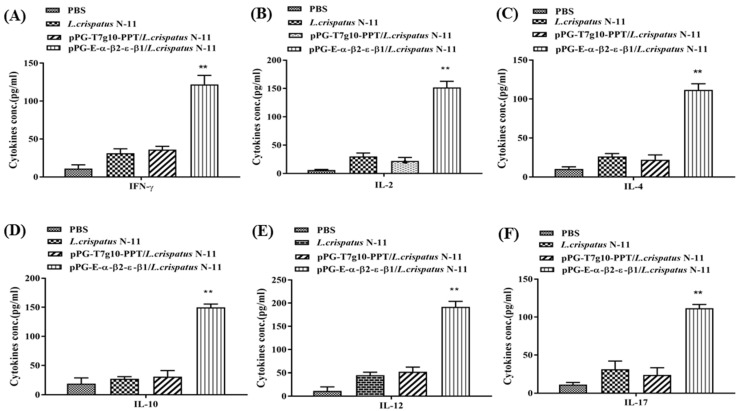
Detection of cytokine levels in the serum of pPG-E-α-β1-ε-β2/*L. crispatus* N-11 immunized chickens. The levels of cytokines IL-2 (**B**), IL-4 (**C**), IL-10 (**D**), IL-12 (**E**), IL-17 (**F**), and IFN-γ (**A**) in the serum samples (*n* = 3) on day 42 post-immunization were detected using an ELISA kit. The data are presented as the mean ± SD (** *p* < 0.01 as compared with groups pPG-T7g10-PPT/*L. crispatus* N-11 and PBS).

Moreover, the levels of cytokines, including IFN-γ, IL-2, IL-4, IL-10, IL-12, and IL-17, were detected in serum samples collected on day 42 post-immunization, suggesting that significantly higher levels (*p* ˂ 0.01) were found in chickens immunized orally with pPG-E-α-β1-ε-β2/*L. crispatus* N-11 than in the pPG-T7g10-PPT/*L. crispatus* N-11 and PBS groups (Figure 5). These data suggest that the recombinant strain could induce the production of Th1, Th2, and Th17 cellular immune responses in chickens.

### 3.4. Immunohistochemical Assays

**Figure 6:**
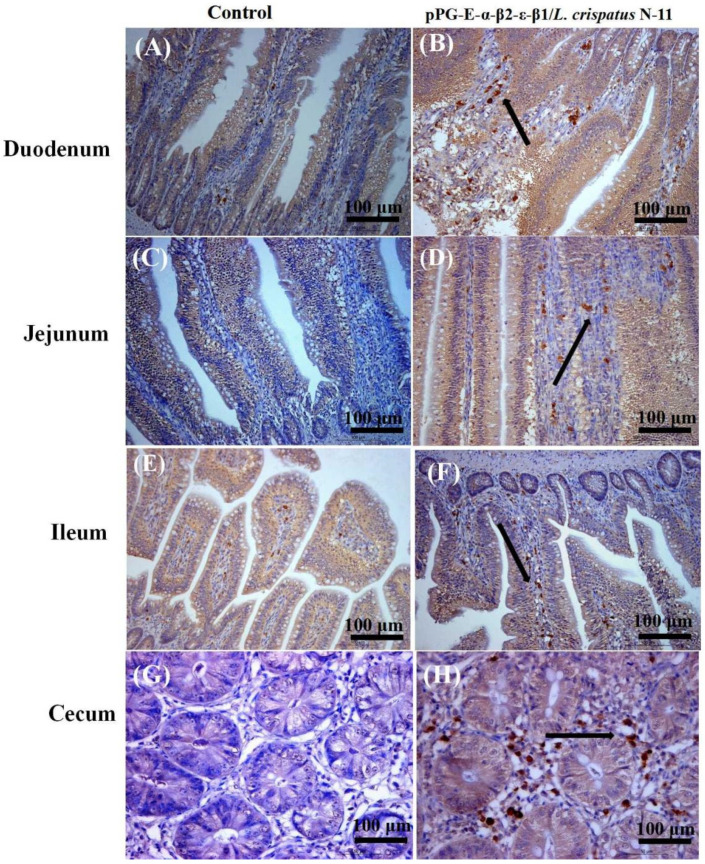
Immunohistochemical assay of chicken immunized with pPG-E-α-β1-ε-β2/*L. crispatus* N-11. The control group (**A**,**C**,**E**,**G**) showed significantly lower IgA positive cells as compared to the pPG-E-α-β1-ε-β2/*L. crispatus* N-11 group (**B**,**D**,**F**,**H**), indicating that pPG-E-α-β1-ε-β2/*L. crispatus* N-11 could effectively enhance mucosal immunity. The black arrows indicate antigen immunoreactivity and the IgA positive cells in the immunized group.

Immunohistochemistry was performed to further evaluate the immune effect of the recombinant strain pPG-E-α-β1-ε-β2/*L. crispatus* N-11. The results indicate that the level of IgA-positive cells were detected in the intestinal samples of chickens immunized with pPG-E-α-β2-ε-β1/*L. crispatus* N-11 (Figure 6A,C,E,G), which were noticeably higher than the control group (Figure 6B,D,F,H), providing evidence that immunization with the recombinant strain confers the ability to enhance mucosal immunity.

### 3.5. Lymphocyte Proliferation

**Figure 7:**
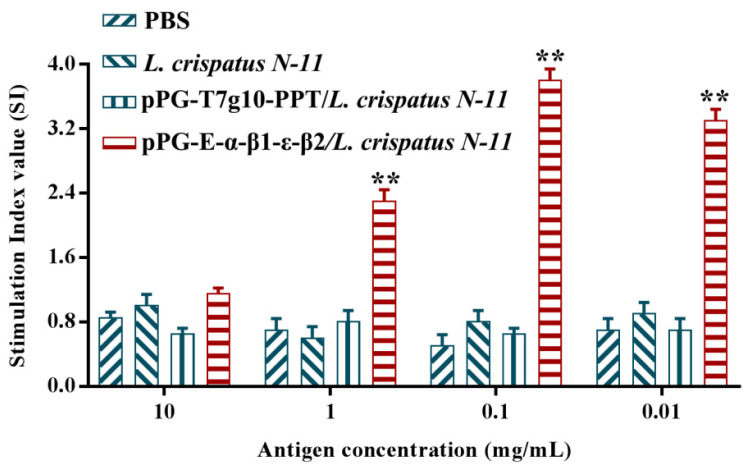
Lymphocyte proliferation. Spleen lymphocytes of chickens (*n* = 3) in the pPG-E-α-β1-ε-β2/*L. crispatus* N-11, pPG-T7g10-PPT/*L. crispatus* N-11, *L. crispatus*, and PBS groups were collected on day 42 post-immunization and restimulated with the α-β2-ε-β1 fusion protein. Then lymphocyte proliferation was evaluated using the MTT assay. The bars symbolize the mean ± standard error in each group. ** *p* < 0.01.

On day 42 post-immunization, the spleen lymphocytes of chickens in each group were isolated and restimulated with the purified fusion protein of *C. perfringens,* α-β2-ε-β1. The MTT assay was used to detect lymphocyte proliferation. The result shows that a significant increase in lymphocyte proliferation was observed in the pPG-E-α-β2-ε-β1/*L. crispatus* N-11 group, compared to the pPG-T7g10-PPT/*L. crispatus* N-11, *L. crispatus* N-11, and PBS groups (Figure 7), suggesting the production of a cellular immune response.

### 3.6. Efficacy of the Probiotic Vaccine against α-β1-ε-β2 Toxin

On day 42 post-immunization, a challenge test was performed. Each group was challenged with a lethal oral dose of mixed natural toxins of type A and type B *C. perfringens* combined with oral viable bacteria to estimate the protection efficacy of the engineered strain Ppg-E-α-β1ε-β2/*L. crispatus* N-11. No deaths were found in the experimental chickens orally immunized with recombinant *L. crispatus*, indicating that the orally administered recombinant *Lactobacillus crispatus* could provide protection against the lethal dose of *C. perfringens* toxin and the attack of live bacteria. In contrast, all of the chickens in the pPG-T7g-10-PPT/*L. crispatus* N-11, non-recombinant *L. crispatus* N-11, and PBS groups died. These results suggest that pPG-E-α-β2-ε-β1/*L. crispatus* N-11 can provide effective protection through oral immunization.

**Figure 8:**
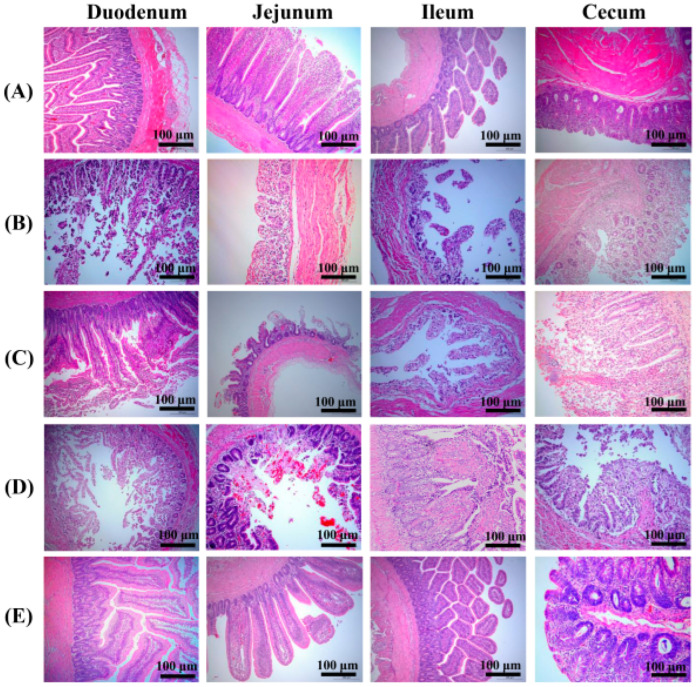
Histopathological changes in immunized chickens after a challenge with the α-β2-ε-β1 fusion protein. Intestinal sections of (**A**) control group without challenge, (**B**) PBS group post-challenge, (**C**) *L. crispatus* N-11 group post-challenge, (**D**) pPG-T7g10-PPT/*L. crispatus* N-11 group post-challenge, and (**E**) pPG-E-α-β2-ε-β1/*L. crispatus* N-11 group post-challenge.

We examined the intestinal tissues of chickens in each group, including the duodenum, jejunum, ileum, and cecum, for histopathological changes. No pathological changes were observed in the control group (Figure 8A). However, severe symptoms and obvious histopathological damage, such as severe disruption of intestinal structural integrity, shortening of villi, and intestinal epithelial necrosis, were observed in the PBS (Figure 8B), *L. crispatus* N-11 (Figure 8C), and pPG-T7g-10-PPT/*L. crispatus* N-11 groups (Figure 8D). Microscopic examination showed an increase in goblet cells, vasodilatation, and hyperemia in the lamina propria of the mucosa. Varying numbers of neutrophil infiltration in the mucosal epithelium and lamina propria, congestion, and a small amount of inflammatory cell infiltration in the submucosa were observed. However, no pathological changes were observed in the pPG-E-α-β2-ε-β1/*L. crispatus* N-11 group, similar to the control group (Figure 8E).

### 3.7. The Effect of Oral Recombinant L. crispatus on the Intestinal Microflora of Chickens

**Figure 9:**
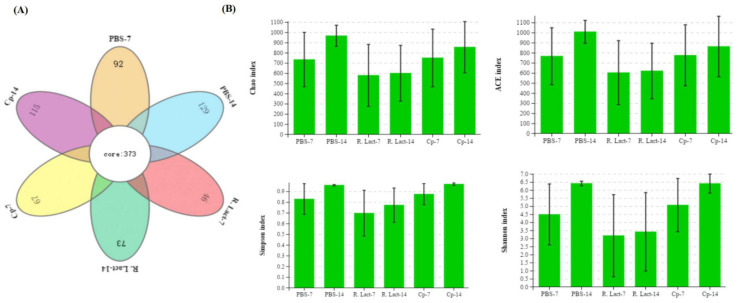
Microbial community configuration of various groups: (**A**) Venn diagram of OTUs. (**B**) Four metrics of analysis, Chao, ACE, Simpson, and Shannon indices, for alpha-diversity among all groups, namely PBS-7, PBS-14 control groups, R. Lact-7, R. Lact-14 recombinant *L. crispatus* groups, and Cp-7 and Cp-14 *C. perfringens* groups.

We analyzed the V3–V4 regions of the bacterial 16S rRNA gene sequence and evaluated the α-diversity indices (Chao, ACE, Shannon, and Simpson indices). The results showed that the gut microbiota of the control, Cp, and R. lact groups differed from each other (Figure 9A,B, *p* < 0.01). Following taxonomic assignment, 373 operational taxonomic units (OTUs) were shared among all groups. Moreover, we found that most OTUs were present individually in different groups (Figure 9A). The microbial diversity in chicken intestinal contents after oral administration of the recombinant *L. crispatus* (R. Lact-7 and R. Lact-14) was measured on days 7 and 14, and was found to be significantly different from the negative control group (PBS-7 and PBS-14) and *C. perfringens* groups (Cp-7 and Cp-14).

**Figure 10:**
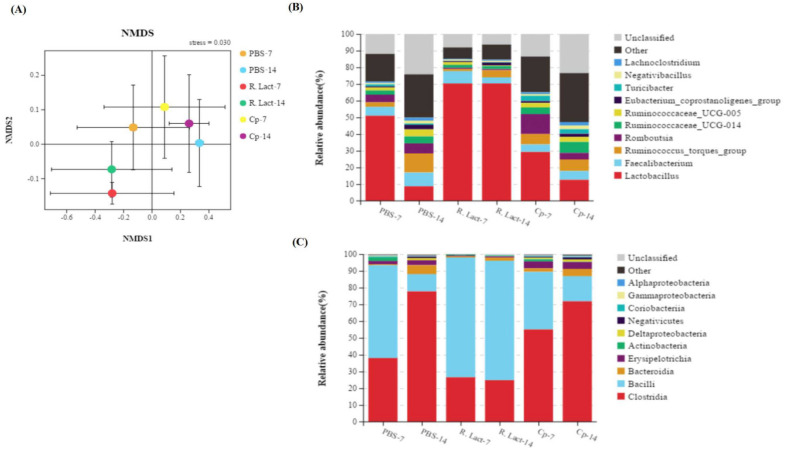
Microbial structure community of different groups. (**A**) Non-metric multidimensional scaling analysis of different groups. (**B**) Comparison of species composition analysis at the genus level. (**C**) Comparison of species composition analysis at the class level.

Table 2’s *L. crispatus* N-11 (R. Lact-7 and R. Lact-14), *C. perfringens* (Cp-7 and Cp-14), and control (PBS-7 and PBS-14) groups were different from each other (Figure 10A). Additionally, in the oral recombinant *L. crispatus* N-11 group, the relative abundance of *Lactobacillus* significantly increased while the abundance of *Eubacterium*, *Lachnoclostridium*, *Romboutsia*, and *Turicibacter* decreased at the genus level as compared to the PBS and Cp groups (Figure 10B). Furthermore, the results revealed a significant difference in composition of microbiota at the class level in the orally administered recombinant *L. crispatus* N-11 group, compared with the control groups; the relative abundance of *Bacillus* increased significantly, whereas that of *Clostridium* decreased (Figure 10C). These results indicate that recombinant *L. crispatus* N-11 pPG-E-α-β2-ε-β1/*L. crispatus* N-11 can play a beneficial role in preventing *C. perfringens* infection.

## 4. Discussion

*C.* *perfringens* is a typical adaptable pathogen in the intestinal tract of livestock and poultry which causes an extensive range of diseases, such as necrotic enteritis, dysentery, and enterotoxemia [25]. The pathogenicity of *C. perfringens* is related to the exotoxin produced by all types of *C. perfringens*, resulting in serious infections [16]. The toxin of *C. perfringens* can increase the cell membrane permeability in poultry, causing cell fluid extravasation, resulting in enterotoxemia and necrotizing enteritis in calves, lambs, and poultry [26]. It is thus critical to develop a vaccine to fight this disease as a preventive treatment, as post-infection treatment is not a viable option [27]. The toxins produced by *C. perfringens* mainly induce the onset of necrotic enteritis and intestinal damage [28]. Therefore, LAB were selected as oral vaccine carriers to deliver antigens, induce the production of intestinal sIgA, and exert a protective effect in the intestinal tract. Gao. et al. used genetically engineered *Lactobacillus casei* (pPG-α/*L. casei* 393) constitutively expressing the toxoid of *C. perfringens*, α-toxin, and evaluated its immunogenicity. They observed a significant increase in sIgA level, with the immune protection rate being 80% [19]. Song et al. constructed an *upp*-deficient *L. casei* strain with a plasmid cross-recombination system, which expressed the α toxin in place of the *upp* gene. This did not necessitate antibiotic screening and provided a reference for the preparation of an oral vaccine against *C. perfringens* [18].

In the current study, as an antigen delivery vector, pPG-E-α-β2-ε-β1/*L. crispatus* N-11, with the EGFP-marked plasmid pPG-E-α-β2-ε-β1, was effectively constructed with the constitutive expression plasmid pPG-T7g-10-PPT. Based on the different types of promoters in the *Lactobacillus* expression system, the system is divided into an inducible expression system and a constitutive expression system. Inducible expression is widely used in the construction of genetically engineered LAB, with lactose, xylose, pH, temperature [29,30,31], and nisin [32] being used as the most common inducers. However, inducible live-vector bacterial expression systems have several limitations. The heterologous protein must be precisely induced by a definite factor; this limits the development of genetically engineered *Lactobacillus* oral vaccines. In the current study, we constructed a constitutive *Lactobacillus* expression system, using the pPG-T7g10-PPT carrier vector [11,22], and applied it to generate genetically engineered *L. crispatus* to express α-β2-ε-β1 toxoids of *C. perfringens.* The results revealed that the fusion protein α-β2ε-β1 could be expressed by recombinant PG-E-α-β2-ε-β1/*L. crispatus* N-11, and the expression was sufficient for the toxoid to be recognized by an anti-toxoid monoclonal antibody. Moreover, the expression of the plasmid toxoid protein on the cell surface of the strain pPG-E-α-β2-ε-β1/*L. crispatus* N-11 was confirmed using laser confocal microscopy. Thus, the membrane-linked, antigen-generating, genetically engineered *Lactobacillus* strain, as an oral vaccine, holds potential to help the host detect antigens and efficiently stimulate antigen-specific immune responses. The ability of genetically engineered LAB to colonize and proliferate in the intestinal tract is a key factor for effective induction of an immune response in the body. Strong adhesion of LAB can effectively improve the immune response of the body [19,29,33]. Therefore, in this study, chickens were used as a model animal to systematically analyze the colonization and dynamic changes of CFDA-SE pPG-E-α-β2-ε-β1/*L. crispatus* N-11 in the intestine. The results indicated that pPG-E-α-β2-ε-β1/*L. crispatus* N-11 could effectively colonize the intestine of chickens and were distributed in the jejunum, ileum, and colon, and likely among other regions. Colonization rates in the jejunum, ileum, and colon of chickens on day 1 after oral administration were high, but gradually decreased, although they remained appreciably high until the 15th day after oral administration. The good colonization capability of pPG-E-α-β2-ε-β1/*L. crispatus* N-11 improved its ability to effectively stimulate the immune response of the body.

An ideal mucosal vaccine must successfully induce both intestinal mucosal and systemic immune responses. sIgA is the dominant antibody on the mucosal surface and plays a significant role in host defense against infections [19,24]. Avian IgA cells are present in the mainstream of intestinal cells, similar to mammals, and produce sIgA, which is then released into the intestinal lumen via trans-epithelial transport and plays a vital role in protection against mucosal infection and maintenance of mucosal homeostasis [34]. Studies have confirmed that utilizing LAB to develop an oral vaccine to deliver foreign antigens can help induce the production of IgA antibodies in animals. Li et al. used auxotrophic *L. casei* and its complementary plasmid to express the non-toxic antigen of Porcine Epidemic Diarrhea Virus (PEDV), and the results showed that it could stimulate the production of sIgA and IgG in mice [35]. Therefore, we endeavored to test the immunogenicity of pPG-E-α-β2-ε-β1/*L. crispatus* N-11 in chickens, immunized orally using ELISA and immunohistochemistry. Our results revealed there was a significant increase in levels of *(p* < 0.01) anti-α-β1-ε-β2 toxoid sIgA-based mucosal immune responses and IgY-based systemic immune responses, indicating that the pPG-E-α-β2-ε-β1/*L. crispatus* N-11 constructed in this study showed better immunogenicity. Furthermore, the IgA content in the chickens immunized with the recombinant strain was significantly higher than that in the control group. The immunohistochemistry results also supported the results of the ELISA antibody detection test.

We found that there was a positive association between cell immunity and lymphocyte proliferation rate. Subsequently, we were able to identify increased spleen lymphocyte proliferation in immunized chickens. Our results indicated that recombinant pPG-E-α-β1-ε-β2/*L. crispatus* N-11 can also induce cellular immune responses. The ratio of T helper 1 (Th1)/(Th2) cells determines the direction of the immune response. According to a previous study [36], *Lactobacillus* can stimulate the progression of innate immune responses and trigger Th1-mediated immune reactions. IFN-γ is mainly produced by Th1 cells and can improve the activity of Th1 cells in promoting cellular immune activity [37]. The Th2-type immune response is marked by the secretion of cytokine IL-4 and production of specific antibodies [38]. In the current study, levels of Th1-type cytokines (IFN-γ, IL-2, and IL-12) and Th2-type cytokines (IL-4, IL-10, and IL-17) in the serum samples of immunized chickens and control groups collected on day 42 post-immunization were analyzed. The results demonstrated significantly higher levels (*p* ˂ 0.01) in chickens immunized orally with pPG-E-α-β1-ε-β2/*L. crispatus* N-11 than in the empty vector PPG-T7g10-PPT/*L. crispatus*, non-recombinant *L. crispatus* N-11, and PBS groups. Additionally, it is worth noting that the concentrations of these cytokines in the serum of chickens in the PPG-T7g10-PPT/*L. crispatus* and *L. crispatus* N-11 groups were higher than those in the PBS group, indicating that the non-engineered probiotics themselves can directly improve the nonspecific immune response. The experimental results show that oral immunization with recombinant *L. crispatus* not only induces a systemic humoral immune response in the body of the animal, but also stimulates the body to produce a cellular immune response. To verify the immune-protective effect of orally administered recombinant *L. crispatus*, we conducted a post-immunization challenge test. The toxin used was a mixture of natural toxins of *C. perfringens* types A and B. The experiment showed that the immunity induced by oral administration of recombinant *L. crispatus* in the vaccine group was able to resist the effect of the natural toxin of *C. perfringens*, with all four chickens tested showing protection, whereas all of the pPG-T7g10-PPT/*L. crispatus* N-11 and PBS control chickens died. Our histopathological observations show that the intestines of the vulnerable groups showed obvious pathological changes compared with that of the chickens orally immunized with recombinant *L. crispatus*. These results indicate that recombinant pPG-E-α-β2-ε-β1/*L. crispatus* N-11 is immunogenic. We also examined the fecal microbiome to explore the effects of oral recombinant *L. crispatus* on the gut microbiota. We analyzed the V3-V4 regions of the bacterial 16S rRNA gene sequence and found a significant difference in the microbial diversity in intestinal feces of the chickens orally administered recombinant *L. crispatus* N-11, compared with the control groups. The relative abundance of *Bacillus* increased significantly, whereas that of *Clostridium* decreased compared to that in the control group.

In conclusion, this study constructed EGFP-marked, genetically engineered *L. crispatus* constitutively expressing the α, β1, ε, and β2 toxoids of *C. perfringens*. Its immunogenicity as an oral probiotic vaccine was explored, and our results showed that pPG-E-α-β2-ε-β1/*L. crispatus* N-11 could effectively induce mucosal, humoral, and cellular immune responses and provide protective effects against a *C. perfringens* challenge. These results demonstrate that the engineered strain is a promising candidate vaccine against *C. perfringens* infections.

## Figures and Tables

**Table 1:** Detection result of intestinal colonization ability of recombinant *L. crispatus*.

Days	% of Total Population		
	Jejunum	Ileum	Colon
1	61.5 ± 1.9	54.4 ± 3.6	57 ± 2.1
4	59.6 ± 2.8	53.6 ± 0.6	58.8± 5.3
7	54.5 ± 3.7	43.7. ± 5.1	45.8 ± 6.6
11	49.5 ± 3.6	36.3 ± 2.4	43.7± 5.3
15	21.5 ± 2.9	17.7± 7.6	19.5 ± 1.6

Each sample was repeated three times, and three chicks (*n* = 3) were sacrificed each time.

**Table 2:** Immunized chickens challenged with *C. perfringens* toxin.

Group	Challenged Dose	Mortality Rate
PBS	1 × LD_100_ + 108 CFU Live bacteria	4/4
*L. crispatus* N-11	1 × LD_100_ + 108 CFU Live bacteria	4/4
pPG-T7g10-PPT/*L. crispatus* N-11	1 × LD_100_ + 108 CFU Live bacteria	4/4
pPG-E-α-β1ε-β2/*L. crispatus* N-11	1 × LD_100_ + 108 CFU Live bacteria	0/4

## Supplementary Materials

The following supporting information can be downloaded at: https://www.mdpi.com/article/10.3390/vaccines10050698/s1, Figure S1: Expression of the protein of interest identified by western blot detection with mouse anti-α/β2/ε/β1 toxins monoclonal antibody; Figure S2: Expression of the protein of interest identified by western blot detection with anti-α toxin polyclonal antibody.

## Abbreviations

Man: Rogosa, and Sharpe (MRS); Luria–Bertani (LB); Horseradish peroxidase (HRP); Carboxyfluoresein diacetate succinimidyl ester (CFDA-SE); sulfate-polyacrylamide gel electrophoresis; Specific pathogen free (SPF); Enzyme-linked immunosorbent assay (ELISA); Immunoglobulin (IgA, IgY). Sodium dodecyl sulfate-polyacrylamide gel electrophoresis (SDS-PAGE).
