# Correction: Khan et al. Oral Immunization of Chickens with Probiotic *Lactobacillus crispatus* Constitutively Expressing the α-β2-ε-β1 Toxoids to Induce Protective Immunity. *Vaccines* 2022, *10*, 698

**DOI:** 10.3390/vaccines12101158

**Published:** 2024-10-11

**Authors:** Mohammad Zeb Khan, Fengsai Li, Xuewei Huang, Muhammad Nouman, Roshna Bibi, Xiaolong Fan, Han Zhou, Zhifu Shan, Li Wang, Yanping Jiang, Wen Cui, Xinyuan Qiao, Yijing Li, Xiaona Wang, Lijie Tang

**Affiliations:** 1 College of Veterinary Medicine, Northeast Agricultural University, Harbin 150030, China; khan2022@126.com (M.Z.K.); yilvwenrou@126.com (F.L.); huangxuewei126@126.com (X.H.); fxl13796687571@163.com (X.F.); zhouhan9659@163.com (H.Z.); shanzhifu@126.com (Z.S.); wanglicau@163.com (L.W.); jiangyanping2017@126.com (Y.J.); cuiwen_200@163.com (W.C.); qiaoxinyuan@126.com (X.Q.); yijingli@163.com (Y.L.); 2 Khyber Medical College, Peshawar 25120, Pakistan; nouman2022@126.com; 3 Department of Boyany, University of Swat, Mingora 19200, Pakistan; bibi2022@126.com; 4 Heilongjiang Key Laboratory for Animal Disease Control and Pharmaceutical Development, Harbin 150030, China

The authors would like to make the following corrections to this published paper [1].

In the original publication, there was a mistake in Figure 8 “Histopathological changes in immunized chickens after a challenge with the α-β2-ε-β1 fusion protein”. The authors misused the same photographed image for the Ileum panel in Figure 8C,D. This was due to the excessive number of pictures produced during the experiment, the presence of visual errors, and the improper preservation of the pictures. The corrected Figure 8 appears as follows.

The authors state that the scientific conclusions are unaffected. This correction was approved by the Academic Editor. The original publication has also been updated.

## Figures and Tables

**Figure 8:**
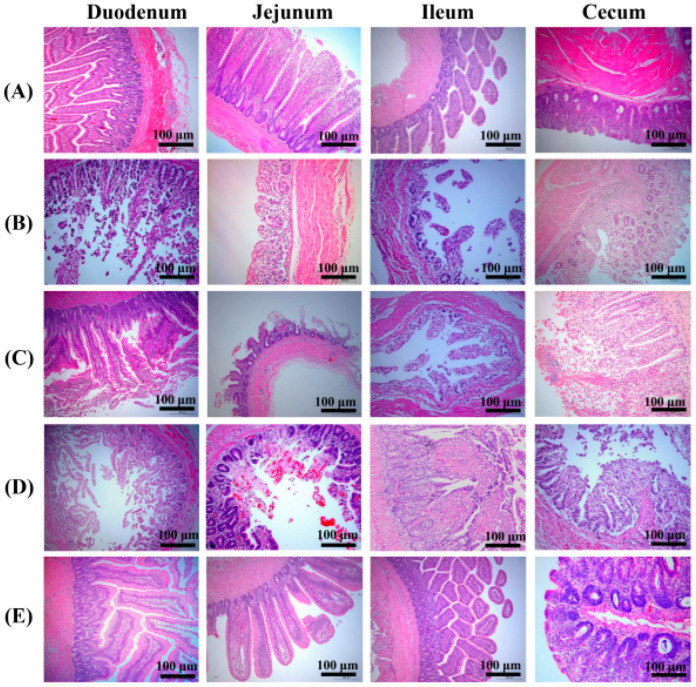
Histopathological changes in immunized chickens after a challenge with the α-β2-ε-β1 fusion protein. Intestinal sections of (**A**) control group without challenge, (**B**) PBS group post-challenge, (**C**) *L. crispatus* N-11 group post-challenge, (**D**) pPG-T7g10-PPT/*L. crispatus* N-11 group post-challenge, and (**E**) pPG-E-α-β2-ε-β1/*L. crispatus* N-11 group post-challenge.
