# Supplementary material for: Oral Immunization of Chickens with Probiotic Lactobacillus crispatus Constitutively Expressing the α-β2-ε-β1 Toxoids to Induce Protective Immunity
Source: Vaccines (Basel). 2022 Apr 29;10(5):698. doi: 10.3390/vaccines10050698 (PMC9147743; doi:10.3390/vaccines10050698)
Supplement: Supplementary file 1 [file vaccines-10-00698-s001.zip › vaccines-1688988-supplementary.pdf]

Original electrophoresis and blotting figures

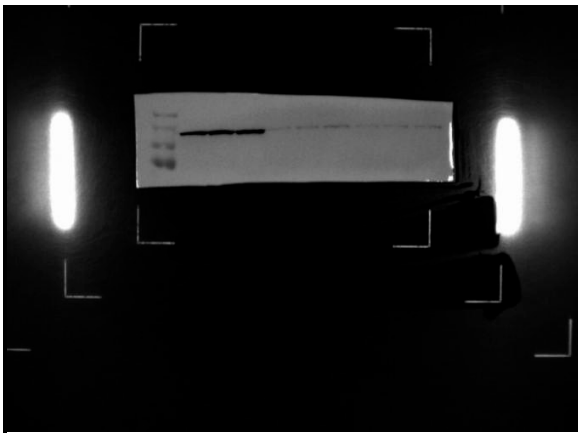

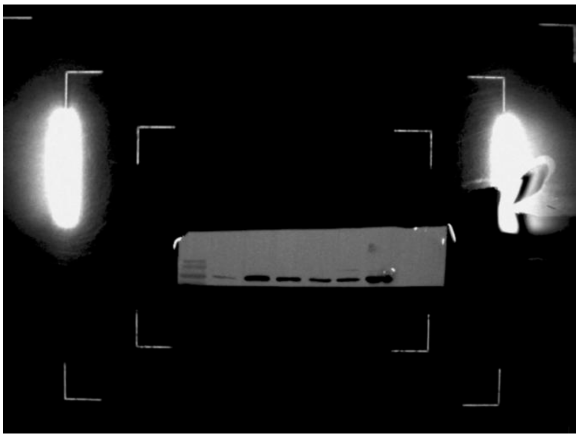

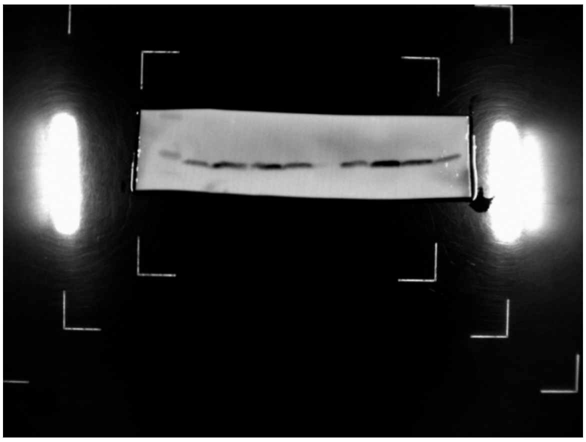

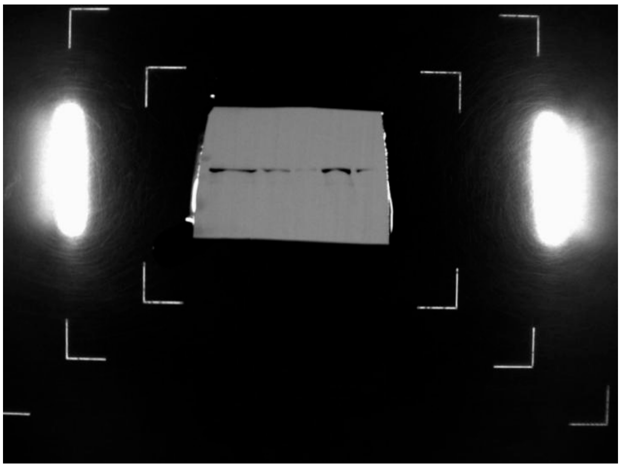

Figure S1: Expression of the protein of interest identified by western blot detection with mouse anti-α/β2/ε/β1 toxins monoclonal antibody.

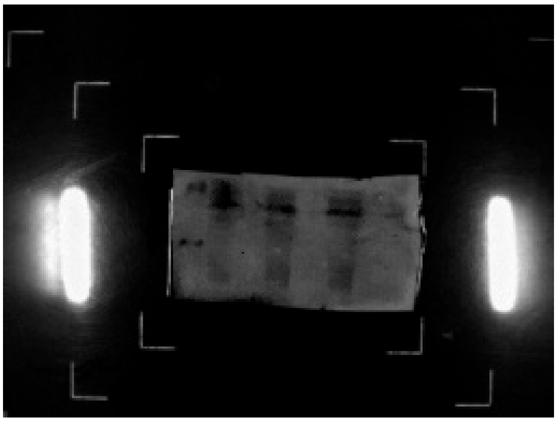

Figure S2: Expression of the protein of interest identified by western blot detection with anti-α toxin polyclonal antibody.
